# *Polygonati Rhizoma* Attenuates Oxidative Stress‐Induced Senescence in Periodontal Ligament Stem Cells

**DOI:** 10.1002/fsn3.71672

**Published:** 2026-06-10

**Authors:** Xinran Feng, Ying An, Fanfan Hou, Qian Guo, Shanshan Cai, Hongbin Xu, Youfu He

**Affiliations:** ^1^ State Key Laboratory of Oral & Maxillofacial Reconstruction and Regeneration, National Clinical Research Center for Oral Diseases, Shaanxi International Joint Research Center for Oral Diseases, The Second Medical Center, School of Stomatology The Fourth Military Medical University Xi'an China; ^2^ Department of Respiratory and Critical Care Medicine The First Affiliated Hospital of Zhengzhou University Zhengzhou China; ^3^ Department of Rhinology First Affiliated Hospital of Zhengzhou University Zhengzhou China; ^4^ Division of Biomedical and Life Sciences, Faculty of Health and Medicine Lancaster University Lancaster UK; ^5^ Longhua Hospital Shanghai University of Traditional Chinese Medicine Shanghai China; ^6^ Department of Cardiology Guizhou Provincial People's Hospital Guiyang Guizhou Province China

**Keywords:** dentistry, network pharmacology, periodontal ligament stem cells, periodontitis, periodontology, *Polygonati Rhizoma*

## Abstract

Periodontitis is increasingly recognized as a non‐communicable chronic oral disease in which sustained oxidative stress and cellular senescence drive low‐grade inflammation and tissue destruction. *Polygonati Rhizoma (PR)*, a traditional Chinese herb widely consumed as a functional food ingredient, is rich in antioxidant polyphenols and polysaccharides, yet its role in modulating senescence of periodontal ligament stem cells (PDLSCs) remains unclear. This study integrated network pharmacology and experimental validation to elucidate the protective effects and mechanisms of *PR* against oxidative stress‐induced PDLSC senescence. Putative *PR* components and targets related to periodontitis and aging were collected to construct a protein–protein interaction (PPI) network, from which 21 core targets were predicted. These included key inflammation‐ and senescence‐related proteins such as TP53, RELA (p65), TNF‐α, TGF‐*β*1, IL‐6, IL‐1*β*, CXCL8, and MMP‐9. Gene Ontology and KEGG enrichment analyses highlighted pathways involved in cellular senescence, NF‐κB signaling, and oxidative stress responses. Molecular docking suggested strong binding affinities between major *PR* constituents and putative core protein targets. In an in vitro oxidative stress‐induced PDLSC senescence model, *PR*‐medicated serum, with Metformin as a positive control, significantly reduced intracellular oxidative stress markers, alleviated cell‐cycle arrest, and downregulated senescence‐associated secretory phenotype (SASP) cytokines. *PR* showed anti‐senescent efficacy comparable to Metformin while exhibiting distinct molecular signatures. These findings indicate that *PR* exerts multi‐component, multi‐target modulation of NF‐κB‐centered inflammatory and senescence pathways in PDLSCs, thereby mitigating oxidative stress‐induced cellular aging. As an edible, functional herb, *PR* may represent a promising dietary adjunct for the prevention and management of periodontitis linked to cellular senescence.

## Graphical abstract

Network pharmacology identified the potential targets and pathways of *Polygonati Rhizoma* (*PR*) against aging‐related periodontitis through PPI, GO/KEGG enrichment, and molecular docking. Experimental validation in a D‐galactose‐induced senescence model of periodontal ligament stem cells showed that *PR*‐medicated serum attenuated cellular senescence and inflammation‐related changes.

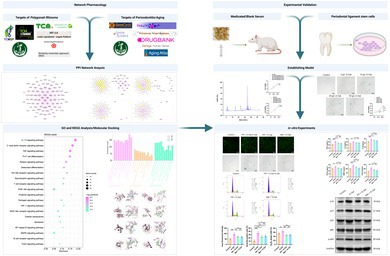

## 1 Introduction

Periodontitis is the primary cause of tooth loss in adults and is characterized by chronic infection that results in the progressive loss of epithelial attachment and alveolar bone (Papapanou et al. 2018). The global prevalence of periodontitis is estimated between 20% and 50% (Czesnikiewicz‐Guzik et al. 2019). In 2010, severe periodontitis was the 6th most prevalent condition, affecting approximately 743 million people worldwide; a figure that surged to 1.1 billion cases by 2019 (Global, Regional, and National Incidence, Prevalence, and Years Lived With Disability for 354 Diseases and Injuries for 195 Countries and Territories, 1990‐2017: A Systematic Analysis for the Global Burden of Disease Study 2017 2018). Conventional treatments primarily aim to control plaque and reduce inflammation, but often fail to restore damaged periodontal tissues fully, leading to inconsistent clinical outcomes (Meng et al. 2022; Sanz et al. 2020). Advances in regenerative medicine, particularly tissue engineering, now offer promising prospects for periodontal regeneration using mesenchymal stem cells (MSCs) (Iwayama et al. 2022; Nguyen‐Thi et al. 2023). Among these, periodontal ligament stem cells (PDLSCs) stand out for their exceptional ability to form cementum and periodontal ligament‐like structures (Nguyen‐Thi et al. 2023; Wen et al. 2024). However, despite progress in cell isolation techniques (Behfarnia et al. 2023; Rad et al. 2022), maintaining cell viability remains a challenge due to issues like cellular senescence and inflammation, which impede the effectiveness of tissue engineering (Lin et al. 2024). As a result, optimizing the use of PDLSCs for periodontal regeneration remains a key focus for ongoing and future research.

Periodontitis is a chronic, multifactorial disease triggered by a dysbiotic biofilm and sustained by an aberrant host response (Kinane et al. 2017). Although chronological aging per se is not an independent risk factor for periodontitis, does not clearly alter plaque microbiology, and has only a limited impact on treatment response (Needleman and Clark 2023), multiple hallmarks of biological aging—notably cellular senescence, stem, progenitor cell exhaustion, and immune‐senescence—are increasingly documented in periodontal tissues and are mechanistically linked to disease susceptibility and progression (Aquino‐Martinez 2023; Baima et al. 2022). Mechanistically, as the first line of defense against bacterial invasion, polymorphonuclear neutrophils (PMNs) undergo a respiratory burst during phagocytosis, releasing large amounts of reactive oxygen species (ROS) that induce oxidative stress, thereby damaging DNA, lipids, and proteins and accelerating cellular senescence (Liguori et al. 2018; Maldonado et al. 2023; Nousis et al. 2023). Senescent cells secrete factors characteristic of the senescence‐associated secretory phenotype (SASP)—including pro‐inflammatory cytokines, chemokines, growth factors, and matrix metalloproteinases (MMPs)—which amplify inflammation and disrupt tissue homeostasis (Aquino‐Martinez 2023; Baima et al. 2022). In parallel, cellular senescence and chronic inflammation can drive stem cell exhaustion, reducing stem cell number and function and impairing MSCs' self‐renewal, multilineage differentiation, and immunomodulatory capacity, thereby hindering periodontal healing and regeneration (Aquino‐Martinez 2023; Baima et al. 2022; Cai et al. 2022; López‐Otín et al. 2023). Against this background, PDLSCs are considered ideal candidates for periodontal regeneration (Shang et al. 2017); delineating their aging process not only clarifies the intersection between periodontitis and biological aging but may also reveal new therapeutic targets.

*Polygonati Rhizoma* (*PR*) was first documented in *Shen Nong's Herbal Classic* during the Eastern Han Dynasty (25–220 ad). It refers to the dried rhizome of *Polygonatum kingianum* Col. et Hemsl., *Polygonatum sibiricum Red*., or *Polygonatum cyrtonema Hua*, as described in the Chinese Pharmacopeia (2020 edition) (National Pharmacopoeia 2020). Modern pharmacological and medicinal chemistry studies have identified various bioactive compounds in *PR*, including polysaccharides, steroidal saponins, triterpenes, alkaloids, lignans, flavonoids, and phytosterols. These compounds exhibit anti‐aging effects through multiple mechanisms, such as anti‐inflammatory, antioxidant, immunoregulatory, mitochondrial protection, and telomerase activation (Zhao et al. 2023). However, the specific biomarkers and biological pathways by which the active components of *PR* mitigate the senescence of PDLSCs remain unclear. Therefore, this study aims to evaluate the potential anti‐senescence effects of *PR* active components on oxidative stress‐induced PDLSCs and to elucidate the underlying molecular mechanisms. To this end, we tested the null hypothesis (*H*_*0*_) that *PR* active components exert no protective effect on the senescence of PDLSCs under oxidative stress.

## 2 Materials and Methods

**FIGURE 1:**
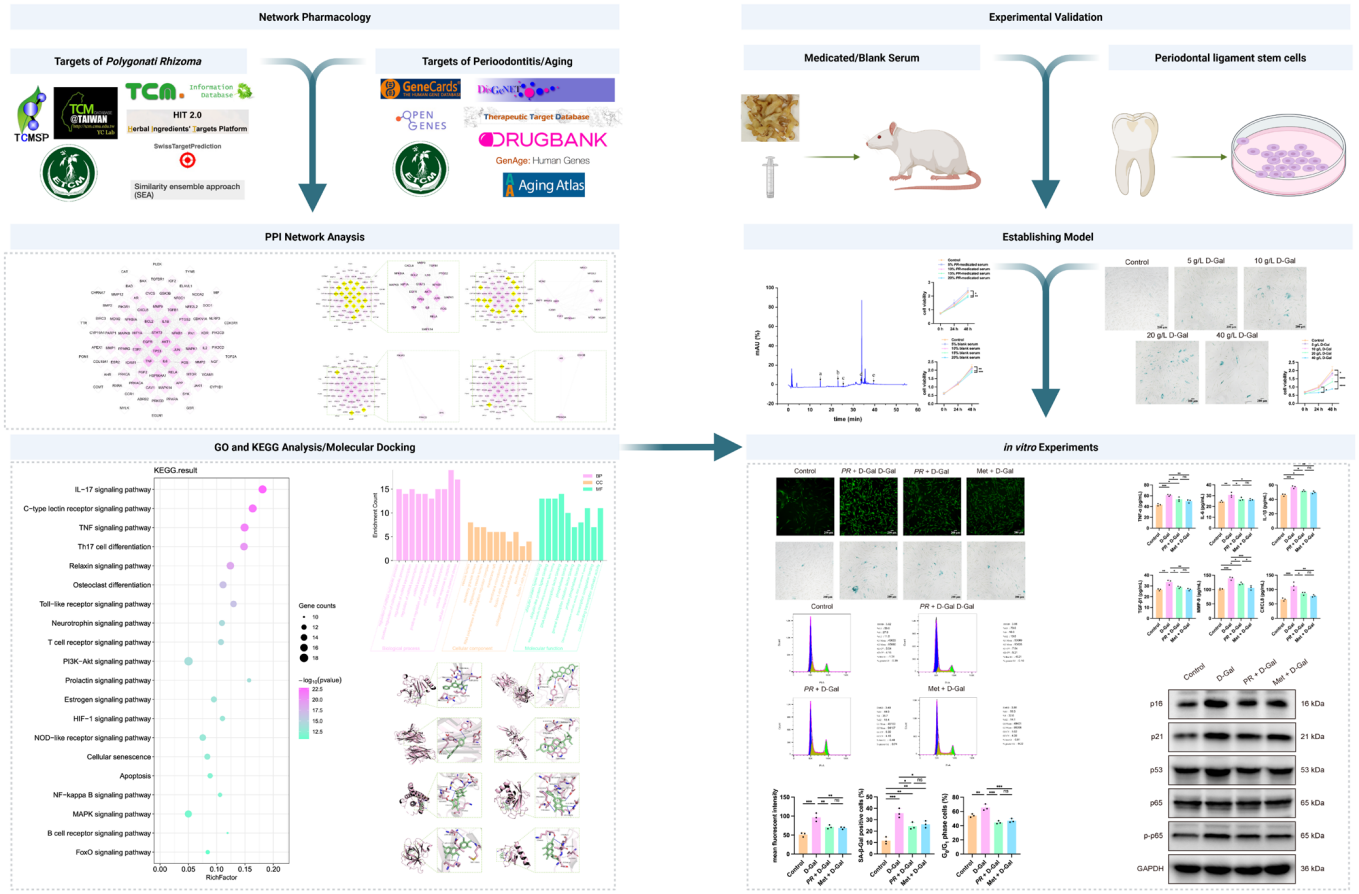
Study methodology flowchart.

This investigation combines network pharmacology with experimental validation to elucidate the anti‐aging mechanisms of *PR* against oxidative‐stress‐induced PDLSC in senescence. A schematic overview of the study workflow is presented in Figure 1.

### 2.1 Network Pharmacology Analysis

#### 2.1.1 Identification of Active Ingredients and Potential Targets of *PR*

The chemical composition of *PR* was obtained from several databases, including Traditional Chinese Medicine Systems Pharmacology Database and Analysis Platform (TCMSP) (Ru et al. 2014), Traditional Chinese Medicine Information Database (TCM‐ID) (Chen et al. 2006), Herbal Ingredients' Targets Platform (HIT) (Ye et al. 2011), The Encyclopedia of Traditional Chinese Medicine (ETCM) (Xu et al. 2019), and Traditional Chinese Medicine Database@Taiwan (TCM Database@Taiwan) (Chen 2011). Active ingredients were identified based on the criteria of oral bioavailability (OB) ≥ 30% and drug‐likeness (DL) ≥ 0.18. Additionally, components that did not meet these criteria but were reported in the literature as active ingredients, quality markers, or compounds with anti‐aging properties or efficacy in treating periodontitis were also included. The active ingredients were then subjected to target prediction using the TCMSP, HIT, SwissTargetPrediction (Daina et al. 2019), and Similarity ensemble approach (SEA) (Keiser et al. 2007) databases. Predicted targets were cross‐referenced with the UniProtKB database (UniProtConsortium 2021) for protein name annotation and corresponding gene symbol identification.

#### 2.1.2 Selection of Aging‐Related Targets of PDLSCs

The GeneCards (Stelzer et al. 2016), Disease Gene Network (DisGeNET) (Piñero et al. 2020), Therapeutic Target Database (TTD) (Zhou et al. 2022), DrugBank database (Wishart et al. 2018), and ETCM databases were queried using the keyword “periodontitis” to identify targets associated with the condition. Furthermore, specialized aging databases, including Aging Atlas (AgingAtlasConsortium 2021), GenAge (Tacutu et al. 2018), and Open Genes (Rafikova et al. 2024), were searched to identify aging‐related targets. By intersecting these two target sets, we identified aging‐related targets relevant to PDLSCs.

#### 2.1.3 Construction of Protein and Protein Interaction (PPI) Network and Enrichment Analysis

The PPI network was constructed using the Search Tool for Retrieval of Interacting Genes/Proteins (STRING) database (Szklarczyk et al. 2021), employing the “Multiple proteins” function, with the species restricted to “ *Homo sapiens* ”, and the minimum required interaction score set to “high confidence (0.700)”. The resulting PPI network was then imported into *CytoScape* 3.10.1 software for visualization. The degree centrality (DC), closeness centrality (CC), and betweenness centrality (BC) of network nodes were calculated using the *cytoHubba* plugin. The Molecular Complex Detection (MCODE) plugin (Bader and Hogue 2003) was then used to cluster the network and identify core sub‐networks and key targets. Gene Ontology (GO) (GeneOntologyConsortium 2015) and Kyoto Encyclopedia of Genes and Genomes (KEGG) (Ogata et al. 1999) enrichment analyses of the key targets were conducted using the *clusterProfiler* package in *R* 4.3.2 software.

#### 2.1.4 Molecular Docking

Protein crystal structures were retrieved from the Research Collaboratory for Structural Bioinformatics Protein Data Bank (RCSB PDB) database (Berman et al. 2000). *PyMOL* open‐source software version 4.6.0 was used to remove water molecules and redundant protein chains. Original ligands were separated to define the center and dimensions of the docking box. The processed structures were then converted to “Protein Data Bank, Partial Charge & Atom Type” (PDBQT) format using *AutoDockTool* (Morris et al. 2009) software version 1.5.7.

The 3D structure of small molecules was obtained from the PubChem database in “Structure‐Data File (SDF)” format and converted to “PDBQT” format using *OpenBabel* (O'Boyle et al. 2011) software version 3.1.1. This conversion involved adding polar hydrogens, assigning charges according to the Merck Molecular Force Field, 1994 parameter set (MMFF94) force field, and performing energy minimization. The active components of *PR* were docked with the core targets using *AutoDock Vina* software version 1.1.2. Interaction patterns were analyzed using Protein‐Ligand Interaction Profiler (*PLIP*) software version 2.2.0, and the results were visualized with *PyMol* open‐source software version 4.6.0.

### 2.2 Verification Experiments

#### 2.2.1 Cell Culture

The experiments involving human tissue samples were approved by the Ethics Committee of the School of Stomatology, China Medical University, Shenyang, China (K2023007). PDLSCs were isolated and identified as described in our previous publications (Feng et al. 2024; Jing et al. 2024). Briefly, healthy premolars or third molars were collected from individuals aged 18–25 years at the university's dental clinic after informed consent. The extracted teeth were rinsed with 5% phosphate‐buffered saline (PBS; Gibco, USA) containing 5% penicillin/streptomycin (PS; Gibco, USA) until blood stains were no longer visible. Periodontal tissues were then scraped from the middle third of the root, cut into small fragments, and distributed evenly into a 6‐well plate containing 2 mL of low glucose‐Dulbecco's Modified Eagle Medium (L‐DMEM; Gibco, USA) supplemented with 20% fetal bovine serum (FBS; Gibco, USA) and 1% PS. The plates were incubated in a humidified atmosphere of 5% CO_2_ at 37°C. The medium was replaced every three days until adherent cell outgrowth was observed under an inverted phase‐contrast microscope (CKX31, OLYMPUS, Japan). Single‐cell suspensions were prepared and diluted to 10 cells/mL, then plated into 96‐well plates with 100 μL of L‐DMEM containing with 10% FBS and 1% PS per well to cultivate individual clones. All monoclonal cell populations were pooled as passage 1 PDLSCs. Cells were sub‐cultured approximately every three days at a 1:3 ratio upon reaching 80% confluence, with passages 3–5 used for subsequent experiments.

#### 2.2.2 Preparation of *PR* ‐Medicated Serum and Blank Serum

The animal experiments were approved by the Ethics Commission of China Medical University, Shenyang, China (Approval No. CMU20231000). Male Sprague–Dawley rats (8 weeks; 180–220 g) were purchased from SPF (Beijing) Biotechnology Co. Rats were housed under standard conditions (22°C–24°C; 60%–70% humidity; 12‐h light–dark cycle) with free access to food and tap water. Twenty rats were randomly allocated (random‐number table) to a control group and a high‐dose *PR* group (20 g/kg). The *PR* group received once daily intragastric administration of *PR*, and the control group received physiological saline (1 mL/100 g), for 7 consecutive days.

On day 7 (2 h after the last administration), rats were anesthetized in an induction chamber with isoflurane (4%–5% for induction in 100% O_2_ at 1.0 L/min) and maintained at 1.5%–2.5% isoflurane via a nose cone. Depth of anesthesia was confirmed by absence of pedal withdrawal and corneal reflexes. While under deep anesthesia, a midline laparotomy was performed, and blood was collected aseptically from the abdominal aorta using a sterile syringe; exsanguination under deep anesthesia served as the method of euthanasia, and bilateral thoracotomy was performed immediately thereafter to ensure death. Body temperature was maintained on a heating pad throughout the procedure. Serum was inactivated at 56°C for 30 min, sterile‐filtered, and stored at −80°C until use.

#### 2.2.3 High Performance Liquid Chromatograph (HPLC)

To prepare the serum samples, 2 mL of each sample was mixed with 6 mL of methanol, vortexed for 10 min, and centrifuged at 18,000 rpm for 10 min at 4°C. The supernatant was collected, evaporated under nitrogen, and reconstituted in 200 μL of 50% methanol. The solution was vortexed, sonicated for 10 min, and centrifuged again at 18,000 rpm for 10 min at 4°C. The supernatant was reserved for analysis.

To prepare the standard solutions, 2 mg each of *β*‐sitosterol, baicalein, kaempferol, myricetin, and diosgenin were accurately weighed and dissolved in 99.8% methanol (*β*‐sitosterol was dissolved in DMSO) to create stock solutions at a concentration of 1 mg/mL. A 20 μL aliquot from each stock solution was combined into a single volumetric flask and diluted to 2 mL with 99.8% methanol (DMSO for *β*‐sitosterol). This mixture was further diluted to prepare a mixed standard solution with a concentration of 10 μg/mL, then filtered through a 0.45 μm membrane for analysis.

Chromatographic separation was performed using an Agilent Poroshell 120 EC‐C18 column (4.6 mm × 150 mm, 5 μm). The mobile phases were acetonitrile (phase A) and 0.1% phosphoric acid (phase B). Gradient elution was conducted as follows: 0–2 min at 90% B; 2–10 min decreasing to 80% B; 10–25 min decreasing to 65% B; 25–35 min decreasing to 5% B; 35–50 min at 5% B; 50–55 min returning to 90% B. The flow rate was 1.0 mL/min, with detection at 260 nm, a column temperature of 30°C, and an injection volume of 10 μL.

#### 2.2.4 Cell Viability

Cell viability was assessed using Cell Counting Kit‐8 (CCK‐8; APExBIO, USA) following the manufacturer's protocol. PDLSCs were seeded in 96‐well plates at a density of 5 × 10^3^ per well and incubated in L‐DMEM with 10% FBS at 37°C. After cell adhesion, the cells were treated with complete medium containing various concentrations of D‐galactose (D‐Gal; 5, 10, 20, and 40 g/L), *PR*‐medicated serum (5%, 10%, 15%, and 20%), and blank serum (5%, 10%, 15%, and 20%). After 0, 24, and 48 h of stimulation, the medium was replaced with 90 μL of L‐DMEM and 10 μL of CCK‐8 solution per well. The plates were incubated at 37°C for 2 h. Absorbance was measured using a microplate reader (Tecan Mechelen, Belgium) at a test wavelength of 450 nm and a reference wavelength of 600 nm. Data were analyzed and plotted using *GraphPad Prism* (version 9.4.0).

#### 2.2.5 Cell Treatment

The following experiments were divided into four groups:

1. Control group: 24 h after plating, the cells were exposed to L‐DMEM with 5% FBS, and 5% blank serum for 48 h.
2. D‐Gal group: 24 h after plating, the cells were exposed to L‐DMEM with 5% FBS, 5% blank serum, and 10 g/L D‐Gal for 48 h.
3. *PR* + D‐Gal group: 24 h after plating, the cells were exposed to L‐DMEM with 5% FBS, 5% *PR*‐medicated serum, and 10 g/L D‐Gal for 48 h.
4. Met + D‐Gal group: 24 h after plating, the cells were exposed to L‐DMEM with 5% FBS, 5% blank serum, 10 g/L D‐Gal, and 100 μM Met for 48 h.

#### 2.2.6 Intracellular Reactive Oxygen Species (ROS)

Intracellular ROS was detected using ROS Assay Kit (S0033S, Beyotime, China). According to the manufacturer's protocol, stimulated cells were washed with PBS and incubated in culture medium with 10 μmol/L DCFH‐DA at 37°C for 20 min. After incubation, the cells were washed three times with PBS and observed under an inverted phase‐contrast microscope (OLYMPUS, Japan).

#### 2.2.7 Senescence‐Associated *β*‐Galactosidase (SA‐*β*‐Gal) Staining

SA‐*β*‐Gal staining was carried out with SA‐*β*‐Gal Staining Kit (Beyotime, China). Stimulated cells were washed with PBS, fixed with 4% paraformaldehyde for 15 min, and washed three times with PBS. The cells were then incubated with SA‐*β*‐Gal staining solution at 37°C without CO_2_ overnight. Blue‐stained cells were observed under an inverted phase‐contrast microscope (OLYMPUS, Japan) and calculated using *ImageJ* software (version 1.8.0). The fraction of senescent cells was scored as the percentage of SA‐*β*‐Gal‐positive cells relative to total cells.

#### 2.2.8 Cell Cycle

Cell cycle analysis was performed using Cell Cycle and Apoptosis Analysis Kit (C1052, Beyotime, China). After cell synchronization and treatment, PDLSCs were washed three times with cold PBS and fixed with 75% ethanol in PBS at −20°C for 12 h. The cells were then washed with cold PBS and stained with 0.5 mL of propidium iodide (PI) at 37°C for 30 min in the dark. Data were analyzed by flow cytometry (Becton Dickinson, USA) and *FlowJo* software (version 10.8.1).

#### 2.2.9 Enzyme‐Linked Immunosorbent Assay (ELISA)

The SASP in the supernatants of stimulated cells was detected using ELISA Kits (Shanghai GuDuoBio Technology Co. Ltd., China). For this analysis, 50 μL of standards and samples, consisting of 10 μL of test samples and 40 μL of sample solution, were added to 96‐well plates. The plates were sealed and incubated with 100 μL of Horseradish Peroxidase (HRP)‐conjugated reagents per well at 37°C for 60 min. After five washes, 50 μL of substrates A and B were added to each well and incubated at 37°C for 15 min in the dark. Following the addition of 100 μL of stop solution, absorbance was measured at 450 nm using a microplate reader (Tecan Mechelen, Belgium). Standard curves and sample concentrations were calculated using linear regression in *Microsoft Excel* software (version 16.80).

#### 2.2.10 Western Blot

Total protein was extracted from stimulated cells and quantified with Bicinchoninic Acid (BCA) Protein Assay Kit (P0012s, Beyotime, China). After extraction, proteins were separated by sodium dodecyl sulfate‐polyacrylamide gel electrophoresis (SDS‐PAGE; GenScript, USA) and transferred to polyvinylidene difluoride membranes (PVDF; Bio‐Rad, USA). The membranes were blocked using 5% nonfat milk and incubated at 4°C with primary antibodies: rabbit anti‐p16 (CST, 1:1000), rabbit anti‐p53 (CST, 1:1000), rabbit anti‐p65 (BOSTER, 1:800), rabbit anti‐p‐p65 (CST, 1:1000), mouse anti‐p21 (SANTA, 1:400), and mouse anti‐GAPDH (SANTA, 1:400). The membranes were then incubated with secondary antibodies (goat anti‐rabbit, BOSTER, 1:2000; goat anti‐mouse, BOSTER, 1:1500) for 1 h at 37°C. Protein bands were visualized using an Odyssey CLX Imaging system (LI‐COR, USA) and analyzed with *ImageJ* software (version 1.8.0).

### 2.3 Statistical Analysis

Each experiment was independently repeated at least three times. Quantitative data are expressed as mean ± standard deviation and analyzed in *SPSS* software (version 26.0). *GraphPad Prism* software (version 9.4.0) was used for statistical analysis and visual representations. When the experimental design involves a single independent variable, overall differences are first assessed with a one‐way ANOVA; when two independent variables are present, a two‐way ANOVA is conducted. If the ANOVA indicates significance, post hoc comparisons are carried out while controlling the family‐wise error rate (FWER). Tukey's post hoc test, which relies on the studentized‐range (*q*) distribution, is used for all pairwise contrasts, whereas Dunnett's post hoc test, based on the multivariate *t* distribution, is applied for “many‐to‐one” comparisons in which each treatment group is evaluated against a common control. All multiplicity‐adjusted *p*‐values were calculated automatically in *GraphPad Prism* 9.4. Statistical significance was set at *p* < 0.05.

## 3 Results

### 3.1 Network Pharmacology Analysis

#### 3.1.1 Active Components and Targets of *PR*

A total of 56 chemical constituents of *PR* were collected through database searches. These constituents were screened using the criteria of OB ≥ 30% and DL ≥ 0.18, with additional information from literature sources. Ultimately, 20 putative active constituents of *PR* were predicted (Table 1), collectively predicted to target 329 different proteins. Notably, pseudoginsenoside F11 was excluded from the analysis due to the absence of predicted targets across four databases.

**TABLE 1:** Bioactive compounds from *Polygonati Rhizoma (PR)*: Physicochemical data, oral bioavailability (OB), and drug‐likeness (DL).

PubChem CID	Compound name	Molecular weight (g/mol)	Molecular formula	OB (%)	DL
222284	Beta‐Sitosterol	414.7	C29H50O	36.91	0.75
10380176	(R)‐Isomucronulatol	302.32	C17H18O5	67.67	0.26
114829	Liquiritigenin	256.25	C15H12O4	32.76	0.18
5281605	Baicalein	270.24	C15H10O5	33.52	0.21
5319422	3′‐Methoxydaidzein	284.26	C16H12O5	48.57	0.24
162818391	(1R,2S,4R,6R,7S,8S,9R,12R,13R,16S)‐6‐[(3S)‐4‐hydroxy‐3‐methylbutyl]‐6‐methoxy‐7,9,13‐trimethyl‐5‐oxapentacyclo[10.8.0.02,9.04,8.013,18]icos‐18‐en‐16‐ol	446.7	C28H46O4	35.12	0.86
928837	(2R)‐7‐hydroxy‐2‐(4‐hydroxyphenyl)‐2,3‐dihydrochromen‐4‐one	256.25	C15H12O4	71.12	0.18
165521	4′,5‐Dihydroxyflavone	254.24	C15H10O4	48.55	0.19
443024	Acanthoside B	580.6	C28H36O13	43.35	0.77
21633072	Pseudoginsenoside FII	801	C42H72O14	22.77	0.19
441207	Digitoxin	764.9	C41H64O13	—	—
15599558	Soyacerebroside II	714	C40H75NO9	—	—
11775688	3‐(butoxymethyl)‐6,7‐dihydro‐5H‐indolizin‐8‐one	221.29	C13H19NO2	—	—
11499272	Kiancdmzgqmial‐uhfffaoysa—	165.19	C9H11NO2	—	—
3013842	3‐Ethoxymethyl‐5,6,7,8‐tetrahydroindolizin‐8‐one	193.24	C11H15NO2	—	—
5280863	Kaempferol	286.24	C15H10O6	41.88	0.24
5281672	Myricetin	318.23	C15H10O8	13.75	0.31
92095	Sarsasapogenin	416.6	C27H44O3	17.41	0.81
9898279	Ginsenoside Rb1	1109.3	C54H92O23	6.24	0.04
99474	Diosgenin	414.6	C27H42O3	80.88	0.81

#### 3.1.2 Acquisition of Potential Therapeutic Targets

**FIGURE 2:**
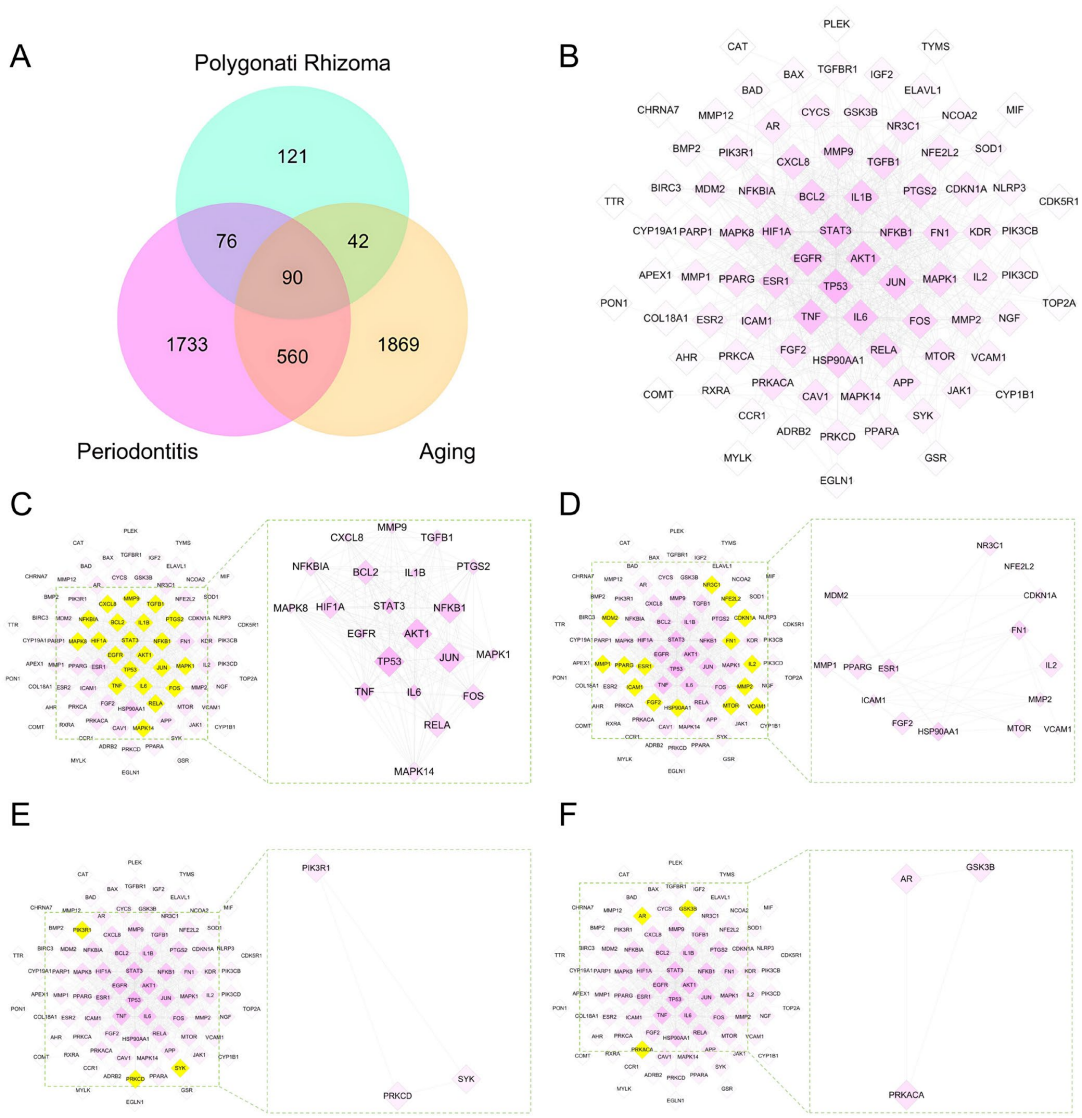
Venn and PPI analyses of *Polygonati Rhizoma (PR)* anti‐aging effects in periodontal ligament stem cells (PDLSCs). (A) Venn diagram of *PR*‐modulated anti‐aging targets in PDLSCs. (B) PPI network; node color intensity ∝ degree centrality. (C) Top‐scoring cluster: Left, full PPI with core targets in yellow; right, enlarged view. (D–F) Additional clusters from the clustering analysis.

Through an extensive database search, we identified 2459 periodontitis‐related targets and 2561 aging‐related targets. By intersecting these with the targets of *PR*'s active constituents, we aimed to identify potential therapeutic targets for *PR* in counteracting the senescence of PDLSCs. This analysis revealed 90 candidate therapeutic targets that may mediate *PR*'s effects on PDLSC senescence (Figure 2A).

#### 3.1.3 Construction of PPI Network and Identification of Core Targets

The therapeutic targets were imported into the STRING database to construct a PPI network. Network topology parameters, including DC, CC, and BC, were calculated using the *cytoHubba* plugin in *CytoScape*. The resulting PPI network contained 86 nodes and 700 edges, with node size determined by decreasing DC, as shown in Figure 2B. Cluster analysis using the *MCODE* plugin identified four network clusters comprising 42 nodes. The average DC, CC, and BC of these clusters were 16.28, 46.78, and 94.09, respectively, significantly higher than the overall network averages. As a result, these 42 nodes were designated as key predicted targets for *PR*'s role in counteracting the senescence of PDLSCs. The highest‐scoring network cluster consisted of 21 nodes, with average DC, CC, and BC values of 34.38, 58.63, and 217.59, respectively (Figure 2C–F, Table 2). For subsequent analyses, putative core targets were defined as those within the highest‐scoring core cluster.

**TABLE 2:** Centrality metrics and MCODE scores for core targets in the PR‐PDLSC PPI clusters.

	Target	MCODE Score	Degree	Closeness	Betweenness
Cluster 1					
	AKT1	14.63	47.00	65.00	659.26
	NFKB1	14.63	39.00	61.25	198.36
	TP53	14.63	50.00	66.75	813.01
	JUN	14.63	40.00	61.92	228.39
	FOS	13.89	30.00	56.42	66.51
	BCL2	13.89	35.00	59.08	214.29
	RELA	13.89	30.00	56.08	130.50
	HIF1A	13.74	35.00	58.75	182.51
	MAPK14	13.27	19.00	50.25	27.76
	TNF	13.23	43.00	63.08	428.41
	NFKBIA	13.18	25.00	53.58	68.98
	PTGS2	12.82	28.00	55.08	46.30
	TGFB1	12.81	27.00	54.92	129.41
	STAT3	12.81	46.00	64.75	341.61
	IL6	12.81	41.00	61.75	253.43
	MAPK1	12.46	30.00	56.92	127.59
	MMP9	12.38	27.00	54.75	82.82
	EGFR	12.20	43.00	63.25	299.38
	MAPK8	12.15	25.00	54.25	61.75
	IL1B	12.09	36.00	59.75	166.35
	CXCL8	12.06	26.00	53.75	42.85
Average		13.25	34.38	58.63	217.59
Cluster 2					
	PPARG	11.12	22.00	52.25	106.42
	IL2	11.10	18.00	49.12	12.05
	FGF2	11.03	21.00	51.08	30.99
	MMP1	11.00	13.00	45.28	0.86
	NR3C1	10.86	17.00	49.58	56.16
	MDM2	10.86	15.00	47.92	2.77
	ESR1	10.81	33.00	58.00	313.84
	FN1	10.76	28.00	55.08	218.22
	HSP90AA1	10.72	32.00	57.83	421.88
	MTOR	10.72	19.00	50.08	42.06
	MMP2	10.37	18.00	49.08	38.58
	CDKN1A	10.37	17.00	49.08	6.82
	ICAM1	10.17	21.00	51.08	14.40
	VCAM1	10.00	11.00	43.78	0.26
	NFE2L2	9.85	17.00	49.42	114.42
Average		10.65	20.13	50.58	91.98
Cluster 3	AR	8.84	15.00	48.33	53.25
	PRKACA	8.00	20.00	49.75	234.21
	GSK3B	7.42	16.00	49.25	43.81
Average		8.08	17.00	49.11	110.42
Cluster 4	PIK3R1	5.41	15.00	48.08	41.79
	PRKCD	5.00	12.00	45.75	31.96
	SYK	5.00	11.00	44.12	22.79
Average		5.14	12.67	45.98	32.18

#### 3.1.4 GO Enrichment Analysis of Key Targets of the PPI Network

**FIGURE 3:**
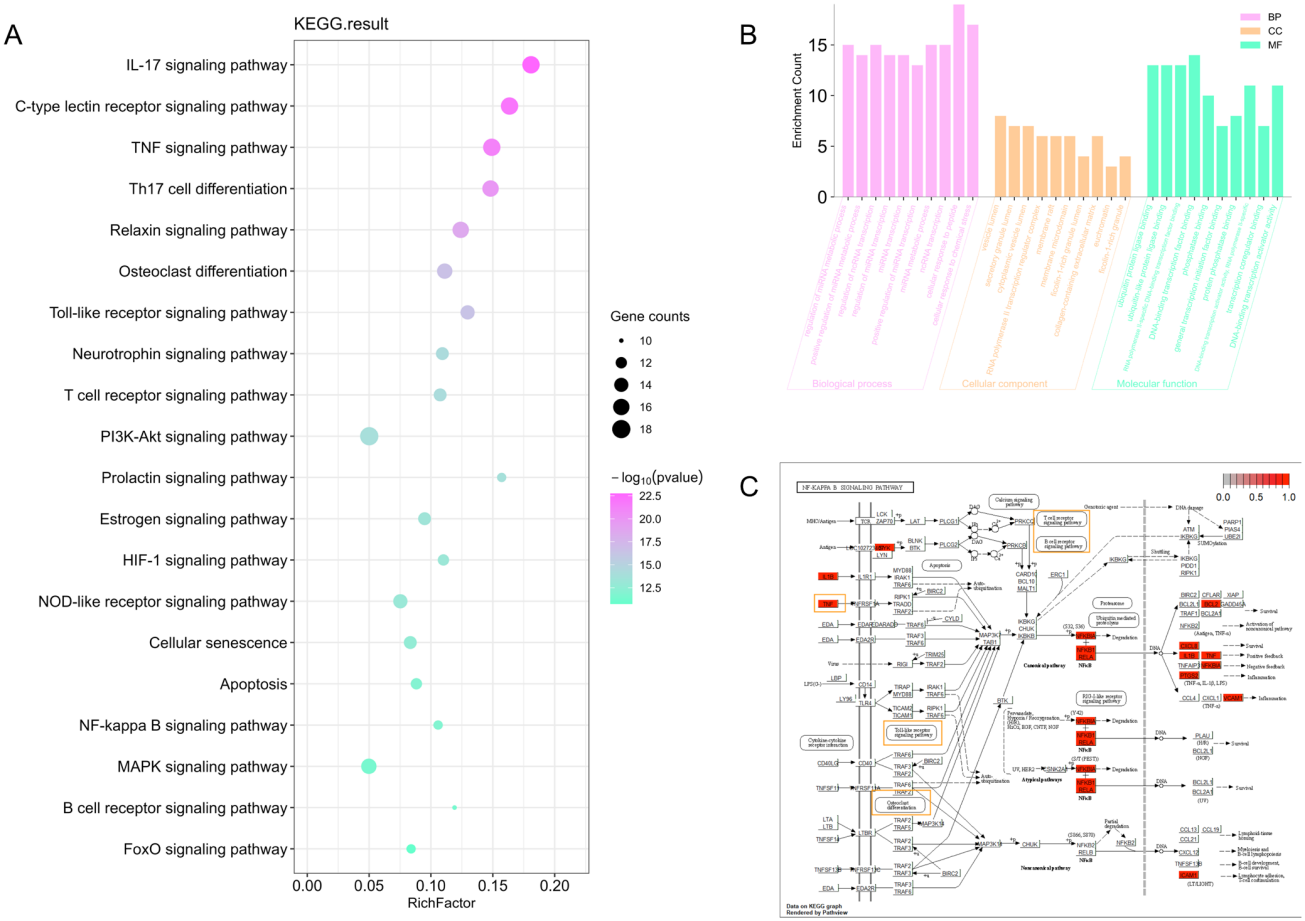
GO/KEGG enrichment and pathway mapping. (A) KEGG enrichment of key targets (top 20). (B) GO enrichment of key targets. (C) Mapping of enriched targets in the NF‐κB signaling pathway.

The 42 key targets of the PPI network were analyzed using the *clusterProfiler* package in *R* 4.3.2, yielding 2054 entries: 1954 in biological processes (BP), 84 in cellular components (CC), and 16 in molecular functions (MF). Figure 3B highlights the top ten significantly enriched entries across these categories. The BP category is enriched in pathways such as miRNA and ncRNA metabolism and the regulation of transcriptional processes. The MF category is associated with activities of ubiquitin‐protein ligases and RNA‐ and DNA‐binding transcription factors. The CC category is related to the structural activities of the cellular vesicle lumen, secretory granule lumen, and other cellular structures.

#### 3.1.5 KEGG Enrichment Analysis of Key Targets of the PPI Network

We conducted KEGG pathway enrichment analysis of the key targets in the PPI network using the *clusterProfiler* package in *R* 4.3.2, identifying 146 pathways with a *p* value < 0.05. This analysis revealed 50 fundamental pathways and 96 disease‐related pathways (Table 3). As shown in Figure 3A, the significantly enriched pathways are primarily involved in signal transduction, cell growth and death, development and regeneration, as well as pathways related to the immune, endocrine, and nervous systems. Cell growth and death‐related pathways include those involved in cellular senescence and apoptosis. Signal transduction‐related pathways encompass the NF‐κB signaling pathway (see Figure 3C) and the TNF signaling pathway.

**TABLE 3:** Significantly enriched KEGG pathways for key targets in the *PR*‐PDLSC PPI network.

Category	Subcategory	ID	Description	Count	*p‐*value
Organismal systems	Immune system	hsa04657	IL‐17 signaling pathway	17	1.80E‐23
Organismal systems	Immune system	hsa04625	C‐type lectin receptor signaling pathway	17	1.14E‐22
Environmental information processing	Signal transduction	hsa04668	TNF signaling pathway	17	6.02E‐22
Organismal systems	Immune system	hsa04659	Th17 cell differentiation	16	1.38E‐20
Organismal systems	Endocrine system	hsa04926	Relaxin signaling pathway	16	2.72E‐19
Organismal systems	Development and regeneration	hsa04380	Osteoclast differentiation	15	2.43E‐17
Organismal systems	Immune system	hsa04620	Toll‐like receptor signaling pathway	14	3.65E‐17
Organismal systems	Nervous system	hsa04722	Neurotrophin signaling pathway	13	5.81E‐15
Organismal systems	Immune system	hsa04660	T cell receptor signaling pathway	13	7.26E‐15
Environmental information processing	Signal transduction	hsa04151	PI3K‐Akt signaling pathway	18	1.22E‐14
Organismal systems	Endocrine system	hsa04917	Prolactin signaling pathway	11	1.49E‐14
Organismal systems	Endocrine system	hsa04915	Estrogen signaling pathway	13	3.76E‐14
Environmental information processing	Signal transduction	hsa04066	HIF‐1 signaling pathway	12	6.86E‐14
Organismal systems	Immune system	hsa04621	NOD‐like receptor signaling pathway	14	8.48E‐14
Cellular processes	Cell growth and death	hsa04218	Cellular senescence	13	2.06E‐13
Cellular processes	Cell growth and death	hsa04210	Apoptosis	12	1.02E‐12
Environmental information processing	Signal transduction	hsa04064	NF‐kappa B signaling pathway	11	1.37E‐12

#### 3.1.6 Molecular Docking

**FIGURE 4:**
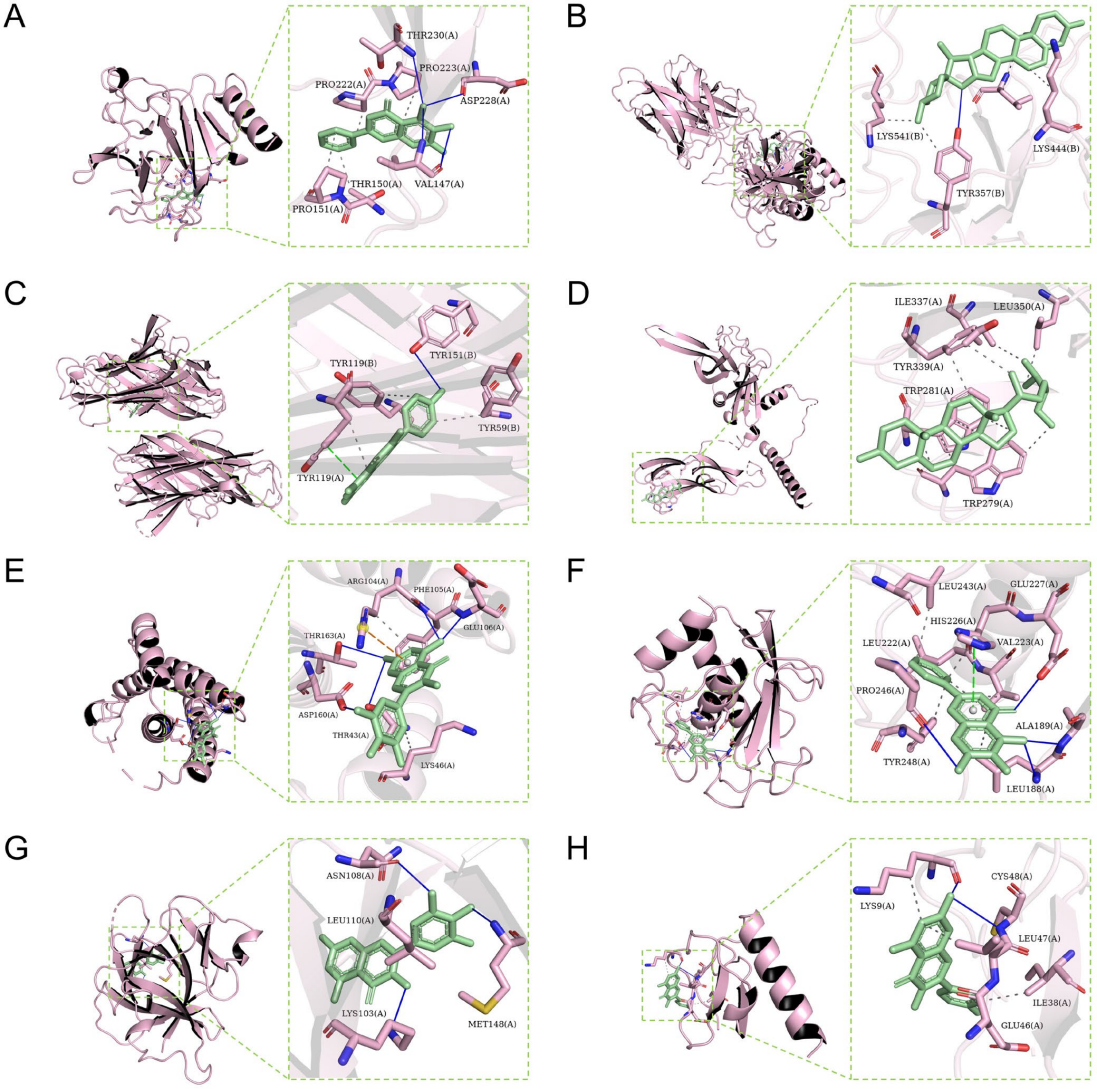
Molecular docking between active *PR* components and core targets. (A) Baicalein‐TP53; (B) Diosgenin‐RELA; (C) Kaempferol‐TNF‐α; (D) *β*‐Sitosterol‐TGF‐*β*1; (E) Myricetin‐IL‐6; (F) Baicalein‐MMP‐9; (G) Myricetin‐IL‐1*β*; (H) Kaempferol‐CXCL‐8.

Through comprehensive analysis, including network clustering, GO and KEGG enrichment studies, and a review of existing literature, we identified tumor protein p53 (*TP53*), transcription factor p65 (*RELA*), tumor necrosis factor‐α (*TNF‐α*), transforming growth factor‐*β*1 (*TGF‐β1*), interleukin‐6 (*IL‐6*), matrix metalloproteinase‐9 (*MMP‐9*), interleukin‐1*β* (*IL‐1β*), and C‐X‐C motif chemokine ligand 8 (*CXCL‐8*) as putative core targets. These targets were used as the basis for molecular docking with the active constituents of *PR*. The docking results, presented in Table 4, indicate an average binding energy of −7.2 kcal/mol for the active components of *PR* against these core targets. The interaction between myricetin and IL‐1*β* exhibited the highest binding energy at −5.5 kcal/mol, whereas baicalein and *MMP‐9* demonstrated the lowest binding energy at −9.7 kcal/mol. This suggests that the components of *PR* may have a high affinity for these key predicted targets, potentially leading to a more stable molecular conformation. Additionally, we thoroughly analyzed the interaction between baicalein and its primary target, confirming that baicalein effectively binds to its target mainly through van der Waals forces and hydrogen bonds, as illustrated in Figure 4.

**TABLE 4:** Docking affinities of *PR* compounds with key protein targets.

PubChem CID	Compound name	Targtet	PDB ID	Affinity (kcal/mol)
5280863	Kaempferol	CXCL8	5D14	−7.1
5281672	Myricetin	IL1B	6Y8M	−5.5
5281672	Myricetin	IL6	1ALU	−6
5280863	Kaempferol	MMP9	5IL2	−9.6
5281605	Baicalein	MMP9	5IL2	−9.7
99474	Diosgenin	RELA	3GUT	−7.3
5281605	Baicalein	RELA	3GUT	−5.8
222284	Beta‐Sitosterol	TGFB1	5VQP	−6.6
5280863	Kaempferol	TNF	2AZ5	−7.3
5281672	Myricetin	TNF	2AZ5	−7.3
99474	Diosgenin	TP53	6GGC	−6.6
5281605	Baicalein	TP53	6GGC	−7.7

#### 3.1.7 Core Biological Network of *PR* Counteracting Senescence in PDLSCs

**FIGURE 5:**
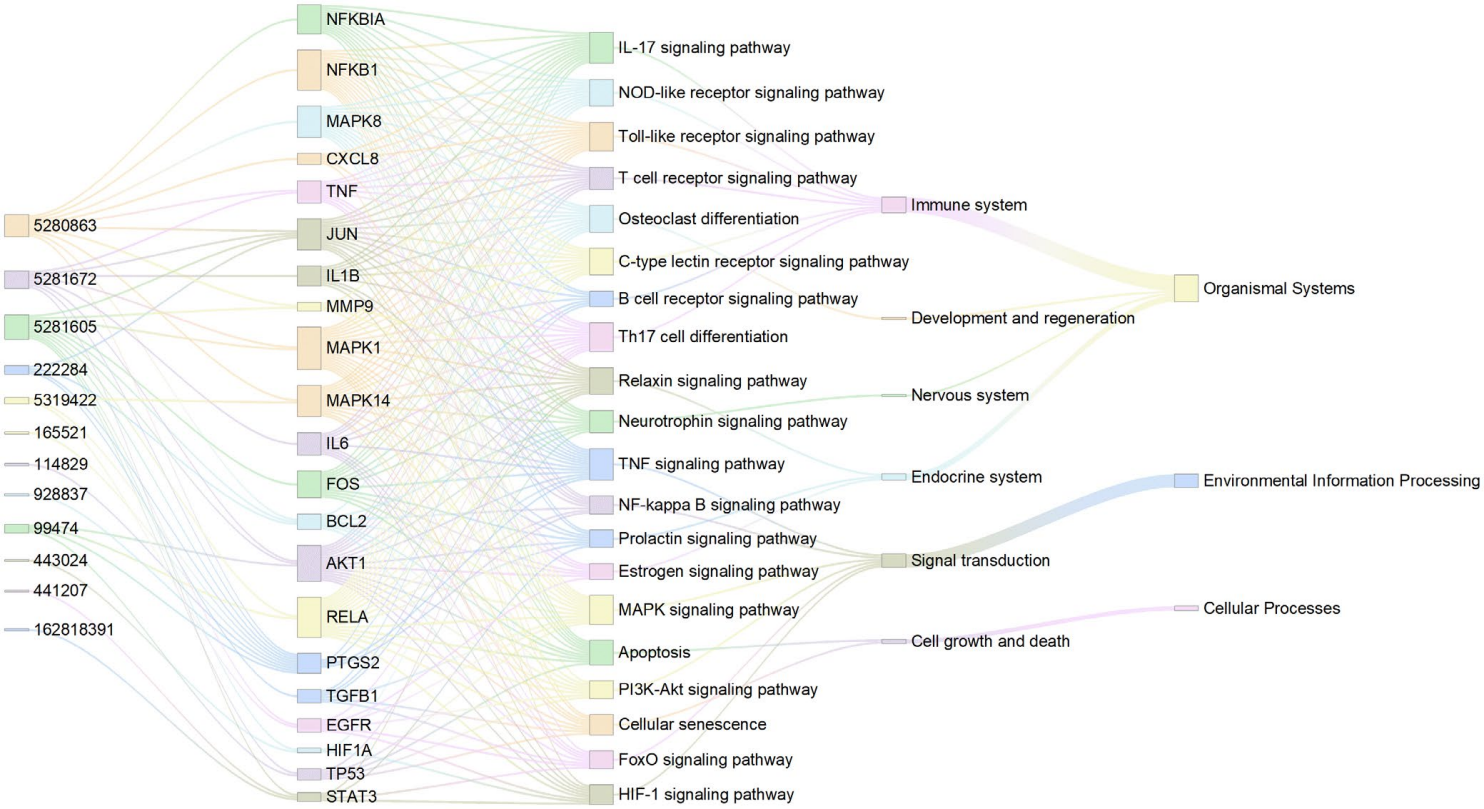
Integrated mechanism by which *PR* counteracts PDLSC senescence.

By mapping the active compounds of *PR* to their core targets and key signaling pathways, we constructed a putative core biological network through which *PR* may counteract the senescence of PDLSCs. As illustrated in Figure 5, *PR* exerts its anti‐aging effects on PDLSCs through a synergistic action on multiple components, targets, and pathways.

### 3.2 Verification Experiments

#### 3.2.1 Identification and Purification of PDLSCs

**FIGURE 6:**
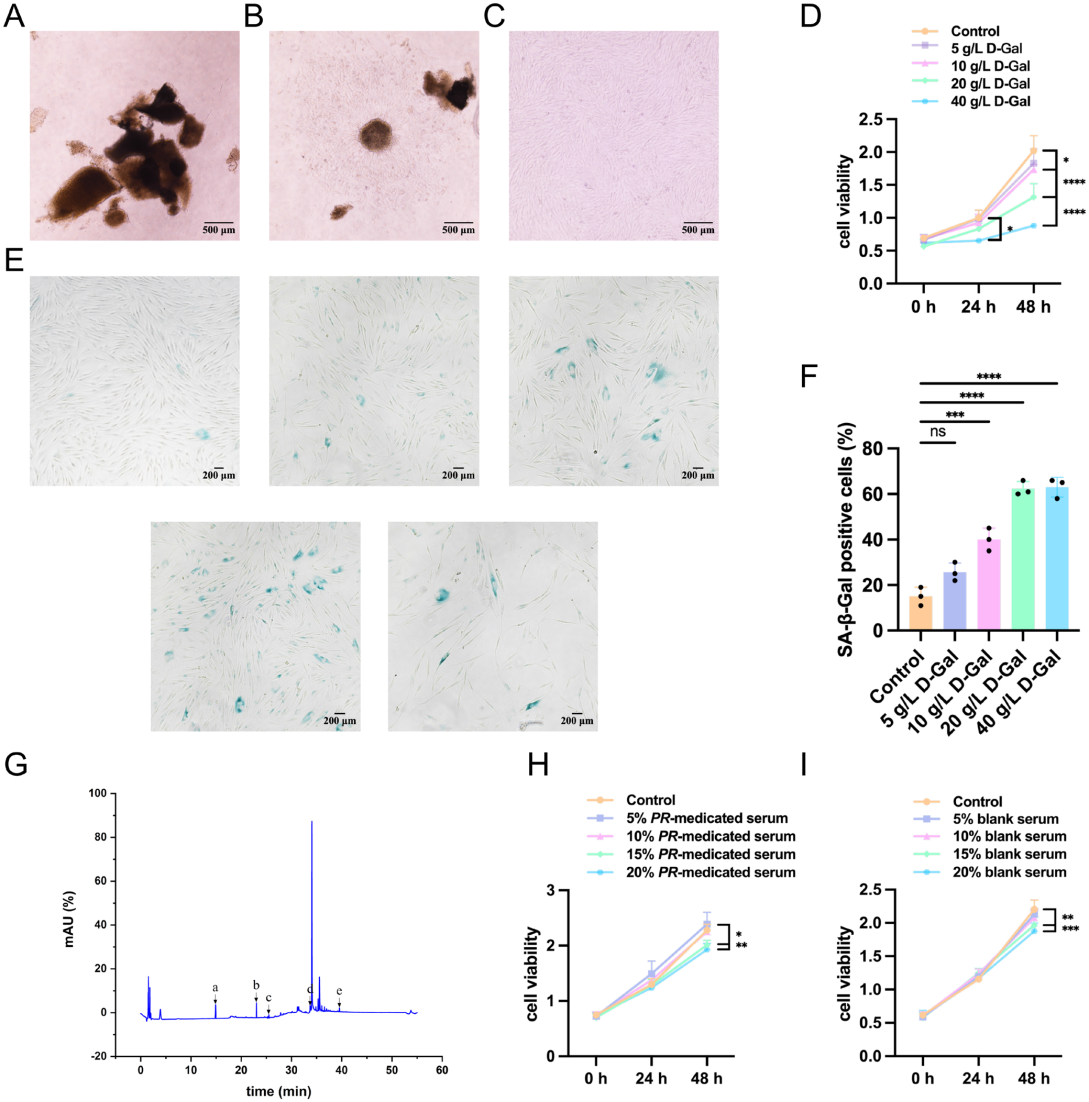
Model establishment and serum characterization. (A) 1 week: Few cells migrate from periodontal ligament tissue block edge (40×). (B) 2 weeks: Radial colony forms around the block (40×). (C) Post‐passage cells retain normal morphology with whirlpool‐like arrangement (100×). (D) PDLSC viability after D‐galactose exposure (CCK‐8). (E) SA‐*β*‐Gal staining after 48 h with 5, 10, 20, 40 g/L D‐galactose; Senescent cells in blue (100×). (F) Quantification of SA‐*β*‐Gal‐positive cells. (G) HPLC chromatogram of PR‐medicated serum: a, myricetin; b, kaempferol; c, baicalein; d, *β*‐sitosterol; e, diosgenin. (H) Effect of *PR*‐medicated serum on viability (CCK‐8). (I) Effect of blank serum on viability (CCK‐8). (Statistics: D, H, I two‐way ANOVA with Dunnett's post hoc; F one‐way ANOVA with Tukey's post hoc; *p < 0.05, **p < 0.01, ***p < 0.001, ****p < 0.0001; ns, not significant).

Periodontal ligament tissues were scraped from the apical third of the root surface. The cells were isolated using the tissue block method and observed under an inverted phase‐contrast microscope after approximately one week. A small number of cells were seen migrating from the edges of the tissue blocks, exhibiting a spindle shape with plump bodies and centrally located round or oval nuclei (Figure 6A). After about two weeks, the cells proliferated and migrated radially, forming radiating cell colonies around the tissue block (Figure 6B). Multiple monoclonal cell lines were combined using limited dilution, and the cultures were expanded. Within 24 h post‐passage, the cells adhered, displayed normal morphology, exhibited good growth, and formed a spiral arrangement (Figure 6C). Third‐generation cells were used for subsequent experiments. The methods and results of cell identification followed the procedures published by our previous research (Jing et al. 2024).

#### 3.2.2 Effect of D‐Gal on PDLSC Activity

The CCK‐8 assay was used to assess the effects of 5, 10, 20, and 40 g/L D‐Gal on the viability of PDLSCs at 0, 24, and 48 h. The results indicated that D‐Gal inhibited PDLSC viability in a time‐ and concentration‐dependent manner. After 0 h, there was no significant difference in PDLSC viability across the groups. However, after 24 h of treatment, PDLSC viability in the 40 g/L D‐Gal group was significantly reduced compared to the control group (*p* < 0.05). After 48 h of treatment, PDLSC viability in the 10, 20, and 40 g/L D‐Gal groups was significantly lower than in the control group (*p* < 0.05, *p* < 0.0001, and *p* < 0.0001, respectively) (Figure 6D).

#### 3.2.3 Effect of D‐Gal on PDLSC Senescence

SA‐*β*‐Gal staining was used to assess the effects of 5, 10, 20, and 40 g/L D‐Gal on PDLSC senescence after 48 h of treatment. The results showed no significant difference in the SA‐*β*‐Gal positivity rate between the 5 g/L D‐Gal group and the control group after 48 h. However, the SA‐*β*‐Gal positivity rates in the 10, 20, and 40 g/L D‐Gal groups were significantly increased (*p* < 0.001, *p* < 0.0001, and *p* < 0.0001, respectively) in a concentration‐dependent manner. Notably, in the 40 g/L D‐Gal group, where cell growth was inhibited, the total number of cells was significantly reduced, yet the SA‐*β*‐Gal positivity rate continued to increase (Figure 6E,F).

#### 3.2.4 Determining the Blood‐Entry Components of *PR* ‐Medicated Serum

HPLC was used to analyze the components of *PR‐*medicated serum. The results revealed that the average retention times for the standards myricetin, kaempferol, baicalein, *β*‐sitosterol, and diosgenin were 15.215, 25.118, 23.815, 34.041, and 39.592 min, respectively. The corresponding peak wavelengths were 270, 360, 254, 210, and 203 nm. By comparing these retention times with the reference standards, it was confirmed that myricetin, kaempferol, baicalein, *β*‐sitosterol, and diosgenin were present in the *PR*‐medicated serum. The overall chromatographic profile, including peak numbers and resolution, indicated that the experimental conditions were optimal, with high stability and reproducibility (Figure 6G).

#### 3.2.5 Impact of *PR* ‐Medicated Serum on PDLSC Activity

The CCK‐8 assay was used to assess the effects of 5%, 10%, 15%, and 20% *PR‐*medicated serum on PDLSC viability at 0, 24, and 48 h. At 0 h, there was no significant difference in PDLSC viability among the groups. After 24 and 48 h of treatment, the 5% *PR‐*medicated serum group exhibited the highest PDLSC viability, although the difference was not statistically significant. After 48 h of treatment, the viability of PDLSCs in the 15% and 20% *PR‐*medicated serum groups was significantly reduced compared to the control group (*p* < 0.05 and *p* < 0.01, respectively) (Figure 6H).

#### 3.2.6 Impact of Blank Serum on PDLSC Activity

The CCK‐8 assay was used to assess the effects of 5%, 10%, 15%, and 20% blank serum on PDLSC viability at 0, 24, and 48 h. The results indicated that after 0 and 24 h of blank serum treatment, there were no significant differences in PDLSC viability among the groups. However, after 48 h of treatment, the viability of PDLSCs in the 15% and 20% blank serum groups was significantly lower compared to the control group (*p* < 0.01 and *p* < 0.001, respectively) (Figure 6I).

#### 3.2.7 Intracellular Oxidative Stress of PDLSCs

**FIGURE 7:**
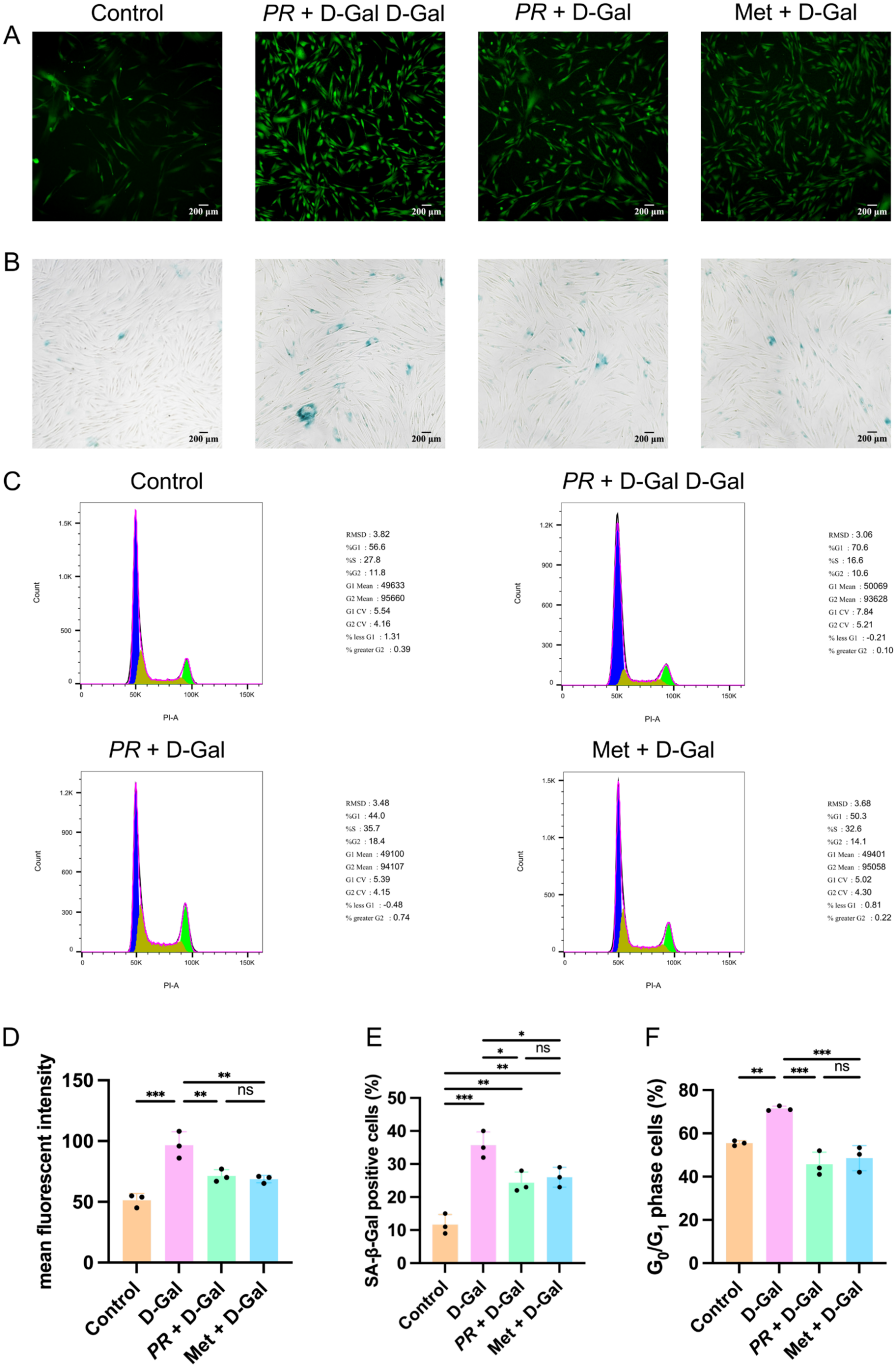
Senescence phenotyping. (A) ROS staining (green intensity reflects ROS; 100×). (B) SA‐β‐Gal staining (senescent cells in blue; 100×). (C) Cell‐cycle distribution by flow cytometry. (D) Mean fluorescence intensity. (E) SA‐β‐Gal‐positive rate. (F) G_0_/G_1_ fraction. (One‐way ANOVA with Tukey's post hoc for D‐F; significance as in Figure 6).

ROS staining was used to assess oxidative stress in PDLSCs following the experimental grouping. The results indicated that ROS levels in the D‐Gal group were significantly higher than in the control group (*p* < 0.001). However, ROS levels in both the *PR‐medicated* serum + D‐Gal group and the Met + D‐Gal group were significantly lower than those in the D‐Gal group (both *p* < 0.01). There was no significant difference in ROS levels between the *PR*‐medicated serum + D‐Gal group and the Met + D‐Gal group (Figure 7A,D).

#### 3.2.8 Senescence of PDLSCs

SA‐*β*‐Gal staining was utilized to assess PDLSC senescence following the experimental grouping. The results showed that the SA‐*β*‐Gal positivity rate in the D‐Gal group was significantly higher than that in the control group (*p* < 0.001). However, the SA‐*β*‐Gal positivity rates in both the *PR*‐medicated serum + D‐Gal group and the Met + D‐Gal group were significantly lower than those in the D‐Gal group (both *p* < 0.05). There was no significant difference in the SA‐*β*‐Gal staining positivity rate between the *PR‐*medicated serum + D‐Gal group and the Met + D‐Gal group (Figure 7B,E).

#### 3.2.9 Cell Cycle of PDLSCs

Flow cytometry was used to analyze the cell cycle distribution in PDLSCs following the experimental grouping. The results showed that the proportion of cells in the G_0_/G_1_ phase was significantly higher in the D‐Gal group than in the control group (*p* < 0.01). Conversely, the proportion of G_0_/G_1_ phase cells in both the *PR‐*medicated serum + D‐Gal group and the Met + D‐Gal group was significantly lower than that in the D‐Gal group (both *p* < 0.001). No significant difference in the proportion of G_0_/G_1_ phase cells between the *PR*‐medicated serum + D‐Gal group and the Met + D‐Gal group (Figure 7C,F).

#### 3.2.10 The Protein Secretion Levels of SASP

**FIGURE 8:**
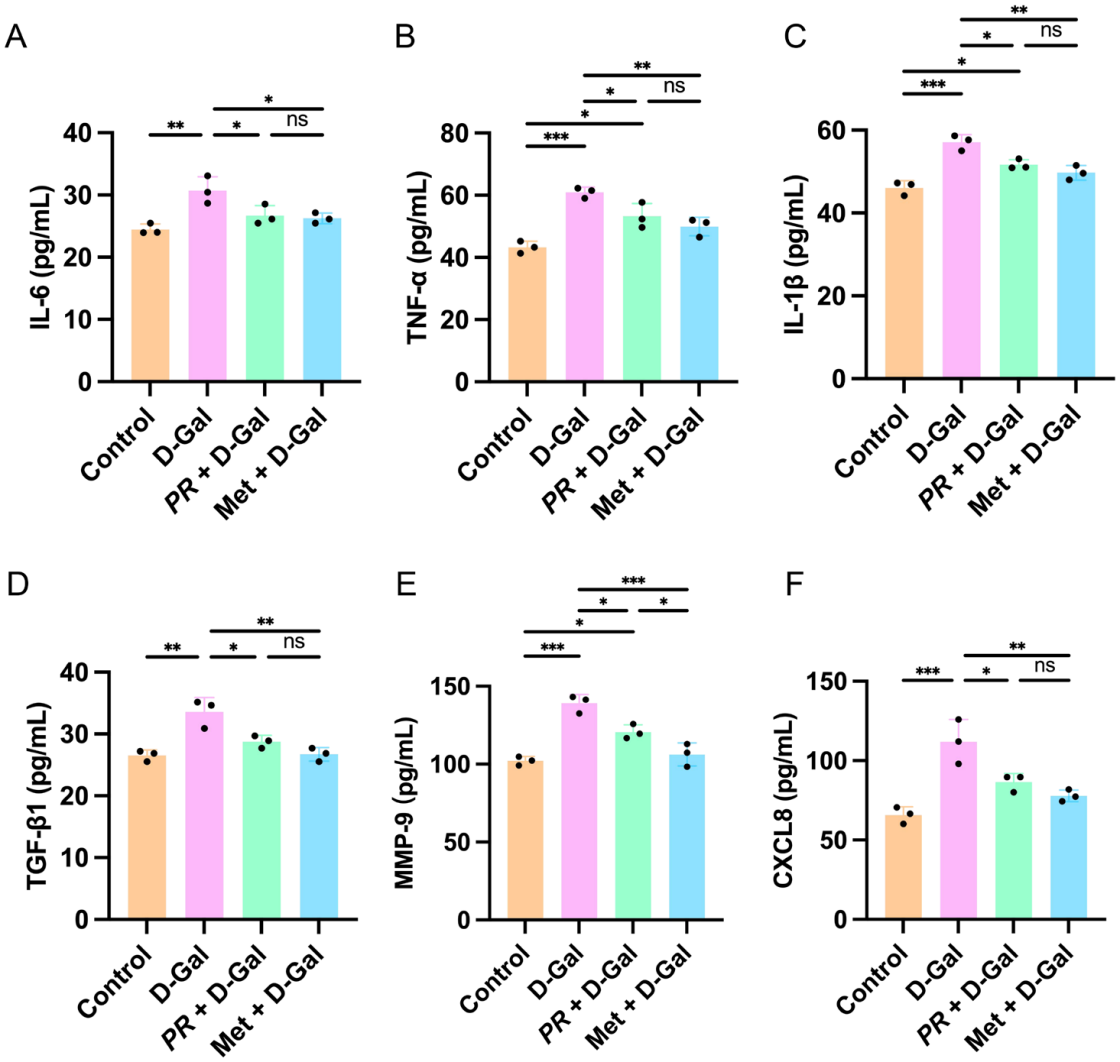
ELISA of senescence‐associated secretory phenotype factors. Secretion levels of (A) TNF‐α, (B) IL‐6, (C) IL‐1β, (D) TGF‐β1, (E) MMP‐9, (F) CXCL‐8. (One‐way ANOVA with Tukey's post hoc; significance as in Figure 6).

ELISA was used to measure the protein secretion levels of pro‐inflammatory factors (TNF‐α, IL‐6, and IL‐1*β*), growth factor TGF‐*β*1, the matrix metalloproteinase MMP‐9, and the chemokine CXCL‐8 within the SASP, following the experimental grouping. The D‐Gal group exhibited significantly higher protein secretion levels of TNF‐α, IL‐6, IL‐1*β*, TGF‐*β*1, MMP‐9, and CXCL‐8 than the control group (*p* < 0.001, *p* < 0.01, *p* < 0.001, *p* < 0.01, *p* < 0.001, and *p* < 0.001, respectively). In the *PR‐*medicated serum + D‐Gal group, the secretion levels of these proteins were significantly lower than in the D‐Gal group (all *p* < 0.05). Similarly, in the Met + D‐Gal group, the protein secretion levels were significantly lower than in the D‐Gal group (*p* < 0.01, *p* < 0.05, *p* < 0.01, *p* < 0.01, *p* < 0.001, and *p* < 0.01, respectively). Furthermore, the Met + D‐Gal group showed a significantly lower secretion of MMP proteins compared to the *PR‐*medicated serum + D‐Gal group (*p* < 0.05). No significant differences in protein secretion levels were observed between the Met + D‐Gal group and the control group (Figure 8).

#### 3.2.11 The Protein Expression Levels of Molecular Markers Related to Cellular Senescence and the NF‐κB Signaling Pathway

**FIGURE 9:**
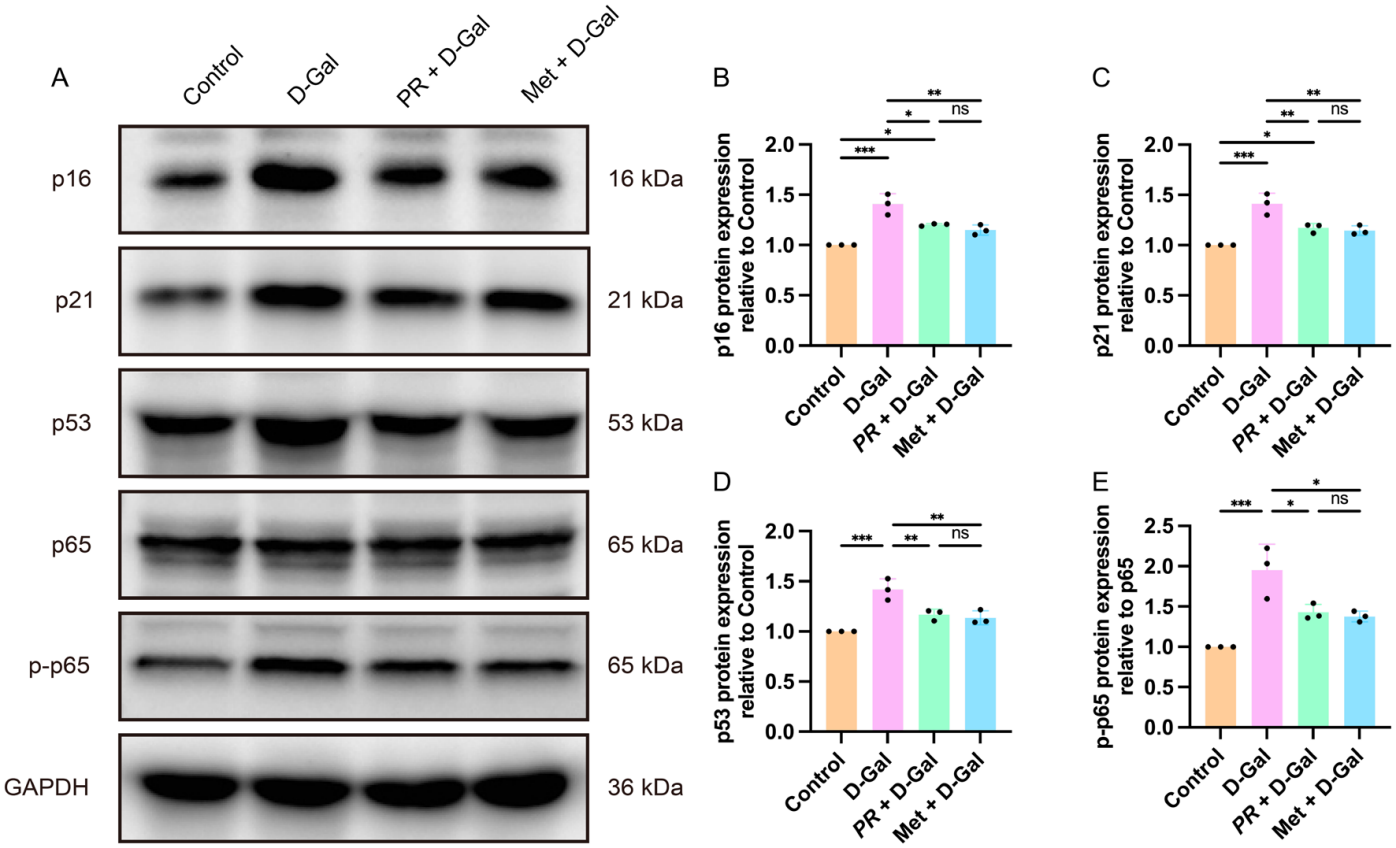
Western blot of senescence markers and NF‐κB signaling. (A) Blot images. (B–E) Densitometry of p16, p21, p53, and phospho‐p65. (One‐way ANOVA with Tukey's post hoc; significance as in Figure 6).

Western blot analysis was used to detect the protein expression levels of cellular senescence markers p16, p21, and p53, as well as key signaling pathway components NF‐κB and p‐NF‐κB, following the experimental grouping. The results demonstrated that the protein expression levels of p16, p21, p53, and p‐NF‐κB were significantly higher in the D‐Gal group compared with the control group (all *p* < 0.001). In the *PR‐*medicated serum + D‐Gal group, the expression levels of these proteins were significantly lower than those in the D‐Gal group (*p* < 0.05, *p* < 0.01, *p* < 0.01, and *p* < 0.05, respectively). Similarly, in the Met + D‐Gal group, protein expression levels were significantly reduced compared with the D‐Gal group (*p* < 0.01, *p* < 0.01, *p* < 0.01, and *p* < 0.05, respectively). No significant differences were observed in the expression levels of these proteins between the Met + D‐Gal group and the *PR‐*medicated serum + D‐Gal group (Figure 9).

## 4 Discussion

Over recent decades, significant efforts have been made in identifying and characterizing stem cells derived from dental tissues, including dental pulp stem cells (DPSCs), stem cells isolated from human pulp of exfoliated deciduous teeth (SHED), PDLSCs, stem cells from apical papilla (SCAPs), and dental follicle progenitor cells (DFPCs) (Zhai et al. 2019). PDLSCs were first identified in 2004 by Seo et al. (Seo et al. 2004) using stem cell markers STRO‐1 (mesenchymal stromal‐cell surface antigen) and cluster of differentiation 146 (CD146, also known as MCAM/MUC18). PDLSCs are notable for their self‐renewal capacity, multipotency (including osteogenic, adipogenic, chondrogenic, and myogenic differentiation), and immunomodulatory properties (Bartold and Gronthos 2017; Hu et al. 2018; Nuñez et al. 2019).

In this study, PDLSCs were successfully isolated from the apical third of the root surface using the tissue block method and limiting dilution method. Our research group previously demonstrated that these cells expressed positive markers for mesenchymal origin (STRO‐1, CD146, CD29 [integrin *β*1], CD90 [Thy‐1], and CD105 [endoglin]) and negative markers for hematopoietic origin (CD45 [protein tyrosine phosphatase receptor type C] and CD34 [hematopoietic progenitor cell antigen CD34]). Additionally, they exhibit the potential for osteogenic and adipogenic differentiation, meeting the identification criteria for PDLSCs (Pan et al. 2017).

D‐Gal is a well‐established senescence‐inducing agent, commonly used to create aging models in vivo and in vitro. At normal concentrations, D‐Gal is oxidized into glucose, but at elevated levels, it converts into aldohexose and hydrogen peroxide, generating large amounts of ROS. Additionally, D‐Gal can be converted into galactitol, which disrupts cellular osmotic pressure, causing swelling and dysfunction, thereby inducing primary cell senescence (Zhang et al. 2009). D‐Gal also reacts with free amino groups in peptides and proteins to form advanced glycation end products (AGEs) (Cui et al. 2006). AGEs can stimulate cells to produce ROS through various pathways, particularly by promoting Nox2 expression via the NADPH oxidase pathway, resulting in a substantial increase in ROS (Zhang et al. 2006). This activates downstream NF‐κB signaling pathways and the expression of SASP‐associated cytokines, inducing secondary cell senescence (Schlett et al. 2023; Songkiatisak et al. 2022; Zhang et al. 2021). Using CCK‐8 assays and SA‐*β*‐Gal staining, we established an in vitro PDLSC senescence model induced by 10 g/L D‐Gal.

The research methodology for serum pharmacology in TCM involves orally administering the herbal medicine or its compounds to animals, followed by blood collection after a set period. The serum is then isolated and used to evaluate efficacy in vitro. This method avoids interference from the physical and chemical properties of crude herbal preparations and reflects the pharmacological effects of digestion, absorption, and biotransformation in the gastrointestinal tract (Bochu et al. 2005). The optimal concentration of medicated serum is determined using “same dosage administration, same blood collection time, varying volume fractions”. Most studies use volume fractions of 5%, 10%, 15%, and 20%. Too low a concentration can slow cell growth, while too high a concentration may lead to rapid cell growth, differentiation, aging, or inhibition of cell proliferation. The optimal concentration often varies depending on the cell type.

In this study, *PR* extract or saline solution was administered to rats, with blood collected and diluted in complete serum medium. CCK‐8 assay results indicated that 5% *PR*‐medicated serum and blank serum were appropriate for PDLSCs, while 15% and 20% concentrations significantly inhibited PDLSC viability. HPLC analysis confirmed the presence of myricetin, kaempferol, baicalein, *β*‐sitosterol, and diosgenin in the *PR*‐medicated serum, validating network pharmacology predictions.

Additionally, Met, a first‐line treatment for type 2 diabetes (Scarpello and Howlett 2008), is known for its role in regulating glucose metabolism (Miller et al. 2013; Xenos et al. 2022), reducing inflammation (Moiseeva et al. 2013), exerting antioxidant activity (Forouzandeh et al. 2014; Polidori and Mecocci 2022), and delaying aging (Boehm and Slack 2006). It can also modulate the production of various SASP factors by inhibiting the NF‐κB signaling pathway (Wiley and Campisi 2021). Studies have shown that 100 μM Met alleviates hydrogen peroxide‐induced PDLSC senescence and restores osteogenic differentiation (Kuang et al. 2020). Based on these findings, 100 μM Met was selected as the positive control for 5% *PR*‐medicated serum in subsequent experiments.

Senescent MSCs display several hallmark changes, including a flattened, enlarged morphology, reduced proliferation and migration, increased ROS levels, elevated SA‐*β*‐Gal activity, and SASP secretion. They also experience cell cycle arrest, impaired multilineage differentiation, and reduced immunomodulatory and paracrine effects (Jiang et al. 2023).

In this study, we found that D‐Gal treatment significantly increased ROS levels, SA‐*β*‐Gal activity, and the proportion of cells in the G_0_/G_1_ phase in PDLSCs. Treatment with *PR*‐medicated serum or Met along with D‐Gal significantly reduced ROS levels, SA‐*β*‐Gal activity, and the proportion of G_0_/G_1_ phase cells, with no significant difference observed between the two treatments. Assays verified that D‐Gal triggered oxidative stress–mediated senescence in PDLSCs in vitro, while *PR*‐medicated serum and Met effectively attenuated it.

As previously mentioned, the active constituents of *PR* have demonstrated potential in reducing inflammation and delaying aging (Zhao et al. 2023). However, the specific biomarkers and biological pathways through which these constituents address PDLSC senescence remain unclear. This study employed a systematic network pharmacology approach to elucidate the mechanisms by which *PR* combats the senescence of PDLSCs, and validated these findings experimentally. Our network pharmacology analysis identified 19 active *PR* components. Molecular docking results indicated strong binding activities between the core targets and key active components, including baicalein, diosgenin, kaempferol, *β*‐sitosterol, and yangmeinin.

Baicalein has been shown to promote the osteogenic differentiation of PDLSCs in both normal (Chen et al. 2017) and inflammatory (Ren et al. 2021) environments by activating the Wnt/*β*‐catenin signaling pathway. It also mitigates the inflammatory response induced by lipopolysaccharide (LPS) in PDLSCs by inhibiting the MAPK signaling pathway (Ren et al. 2021). Additionally, baicalein activates the Nrf2 signaling pathway, reducing high glucose‐induced oxidative stress in human gingival epithelial cells (hGECs) and attenuating alveolar bone resorption in a diabetic periodontitis rat model (Zhu et al. 2020). Encapsulation of baicalein in nanoparticles (Li et al. 2017) and incorporation into chitosan/dioleoylphosphatidylethanolamine‐baicalein nanohydrogels (Guo et al. 2024) has been effective in treating IL‐1*β*‐induced hGECs and diabetic periodontitis rat models respectively, exhibiting significant anti‐inflammatory effects.

Diosgenin was found to reduce the expression and secretion of inflammatory factors, such as *IL‐1β*, *IL‐6*, and *TNF‐α*, in LPS‐induced PDLSCs and inhibit the nuclear translocation of NF‐κB p65 and phosphorylated IκB‐α (Cong et al. 2023). Studies using network pharmacology have also identified kaempferol and *β*‐sitosterol as key active ingredients in periodontitis treatment, showing high binding affinity to B‐cell lymphoma 2 (*BCL2*). In addition, surface‐plasmon‐resonance analyses have confirmed that kaempferol binds directly to human TNF‐α and disrupts the TNF‐α/TNFR1 interface, whereas cellular thermal‐shift assays demonstrate that baicalein directly engages TP53 (Dou et al. 2024; Wang et al. 2022). Sinensis‐angelica, which contains these compounds, has been shown to stimulate the expression of osteogenesis‐related genes and proteins in MC3T3‐E1 cells, providing osteoprotective effects in periodontitis treatment (Chen et al. 2023). HPLC analysis of *PR*‐medicated serum confirmed the presence of populin, kaempferol, baicalein, *β*‐sitosterol, and diosgenin components, supporting the predictions made by network pharmacology analysis.

We identified a total of 8 key targets, including *TP53*, *RELA*, *TNF‐α*, *TGF‐β1*, *IL‐6*, *MMP‐9*, *IL‐1β*, and *CXCL‐8*, that *PR*'s active components may act on to mitigate PDLSC senescence. Molecular docking results revealed a high affinity between these hub genes and *PR*'s active constituents, suggesting their potential therapeutic relevance in combating PDLSC senescence. This study identified a total of 90 targets against PDLSC senescence, of which 42 were classified as key targets, and 21 were identified as core targets for the antagonistic effects of *PR* on PDLSC senescence.

*PR* antagonizes senescence in PDLSCs by targeting molecular markers of cellular senescence, including *TP53* and *RELA* (a key protein in the NF‐κB signaling pathway), as well as various cytokines associated with the SASP. These cytokines include pro‐inflammatory factors such as TNF‐α, IL‐6, and IL‐1*β*, the growth factor TGF‐*β*1, the matrix metalloproteinase MMP‐9, and the chemokine CXCL‐8. IL‐6 is a prominent SASP factor (Cai et al. 2022), while TNF‐α and IL‐1*β* are implicated in many inflammatory diseases and are key targets for anti‐inflammatory therapies (Broderick and Hoffman 2022; Zelová and Hošek 2013). TGF‐*β*1 regulates cell growth and differentiation (Richardson et al. 2023). MMP‐9 is vital for extracellular matrix metabolism (Rashid and Bardaweel 2023). CXCL‐8, also known as IL‐8, promotes tissue repair and infection resistance (Cambier et al. 2023).

Recent animal studies have shown that senescent PDLSCs can contribute to periodontitis development by exacerbating chronic inflammation and periodontal tissue destruction through the secretion of SASP factors (Ikegami et al. 2023). Therefore, targeting SASP factors secreted by senescent PDLSCs is an important strategy for adjunctive treatment of periodontitis. ELISA results showed a significant increase in the protein secretion of SASP factors following D‐Gal treatment. In contrast, Met treatment led to a significant decrease in MMP‐9 protein secretion compared to *PR*‐medicated serum, although no significant difference was observed between Met and the control group. Both *PR*‐medicated serum and Met effectively down‐regulated the secretion of SASP proteins, with Met demonstrating a stronger effect.

The regulation of SASP responses occurs at various levels, including transcription, mRNA stability, translation, and extracellular secretion. SASP activation is specifically mediated by innate immune signaling pathways, mechanistic target of rapamycin complex 1 (mTORC1), and transcription factors such as NF‐κB, CREB‐binding protein (CBP), and GATA‐binding protein 4 (GATA4) (Cai et al. 2022) (Songkiatisak et al. 2022).

KEGG pathway enrichment analysis identified several significantly enriched pathways, including cellular senescence and the NF‐κB signaling pathway. Although the NF‐κB signaling pathway may not be the most enriched pathway in network pharmacology studies, it remains a critical regulator of a range of SASP factors. Additionally, p65, a key protein within the NF‐κB signaling pathway, is considered a core target.

NF‐κB plays a crucial role in regulating the activation of inflammatory T cells and proinflammatory factors within innate immunity. It also regulates T‐cell differentiation and effector functions, making it essential for downstream inflammatory and immune‐related signaling pathways (Lawrence 2009; Tak and Firestein 2001). Inhibition of NF‐κB signaling pathway activation has been shown to mitigate the inflammatory response in animal models of chronic periodontitis, reducing alveolar bone destruction (Wang et al. 2021). The NF‐κB signaling pathway is crucial in counteracting aging and limiting periodontitis‐induced damage.

The NF‐κB family comprises c‐Rel, RelA (p65), v‐rel avian reticuloendotheliosis viral oncogene homolog B (RelB), NF‐kB1 (p50) and NF‐kB2 (p52). Notably, the phosphorylation of RelA (p65) is ROS‐dependent and essential for transcriptional activation of target genes downstream of NF‐κB (Lingappan 2018).

Based on network pharmacology findings, it is hypothesized that PDLSC senescence is mediated through the NF‐κB pathway through promotion of SASP factor secretion. Western Blot analyses revealed a significant increase in protein expression levels of cellular senescence markers and NF‐κB pathway components following D‐Gal treatment. However, these protein levels were significantly reduced by treatment with *PR*‐medicated serum or Met, particularly when administered concurrently with D‐Gal. Additionally, Met significantly reduced NF‐κB signaling protein expression compared to *PR*‐medicated serum. Both *PR*‐medicated serum and Met effectively downregulated markers associated with cellular senescence and the NF‐κB signaling pathway, with Met showing a more pronounced effect.

This study combines network pharmacology and experimental validation to explore the mechanisms by which *PR* mitigates oxidative stress‐induced senescence in PDLSCs, offering a theoretical foundation for its use in periodontal tissue engineering and periodontitis treatment. However, inherent limitations exist due to the aggregation of heterogeneous datasets in network pharmacology, which may introduce information bias. Incompletely curated compound libraries may miss minor *PR* constituents, leading to false‐negative target predictions, while target repositories biased toward oncology and immunology may overrepresent well‐studied hubs such as *TP53* or *TNF‐α*. Consequently, some of the 21 ‘core’ targets may be over‐ranked, and under‐represented proteins may remain relevant. Additionally, static protein‐interaction graphs cannot capture dynamic, tissue‐specific, or feedback loop interactions, and thus, pathway read‐outs should be considered putative.

To address these issues, we (i) queried five independent databases, (ii) annotated targets using four in silico algorithms, (iii) confirmed compound‐target interactions via molecular docking, and (iv) validated downstream signaling inhibition in senescent PDLSCs through Western blot and ELISA. Despite these efforts, all hypotheses remain provisional and require further validation. Molecular docking, which treats proteins as rigid, can misestimate binding affinities, and direct ligand‐target interaction evidence is limited (Desta et al. 2020; Huang and Zou 2010). Future studies should employ co‐immunoprecipitation to confirm interactions between *PR* components and targets.

Although this study is based on in vitro assays, rodent models have confirmed *PR*'s efficacy against senescence and periodontitis (Huang et al. 2025; Pan et al. 2024; Wu et al. 2024; Zheng 2020). Our next phase will use a ligature‐induced periodontitis mouse model to validate the NF‐κB mechanism and assess pharmacokinetics, dose optimization, and long‐term safety.

Clinically, *PR* could be applied as a host‐modulating gel or nanoparticle spray after scaling‐and‐root‐planing (SRP) to promote inflammation resolution and bone regeneration. It may also be combined with low‐dose chlorhexidine or doxycycline to enhance antibacterial and host‐response effects while mitigating antibiotic resistance. Additionally, it could serve as a mouth rinse or muco‐adhesive patch for early or maintenance therapy. A stepwise translational roadmap is proposed, starting with preclinical studies, followed by a dose‐finding study in a periodontitis model, and concluding with a randomized controlled trial comparing SRP with and without *PR* gel.

## 5 Conclusions

*PR* mitigates oxidative‐stress‐induced senescence in PDLSCs through multi‐component, multi‐target modulation predicted by our network‐pharmacology workflow and experimentally validated. *PR‐medicated* serum reduced oxidative damage, curtailed the SASP via the NF‐κB pathway, and performed comparably to metformin in most readouts, although metformin showed a slightly stronger suppression of SASP proteins. These findings position *PR* as a promising adjunct therapy for delaying PDLSC senescence and advancing periodontal tissue‐engineering strategies, while the network pharmacology predictions also illuminate new putative avenues for anti‐aging interventions in periodontitis.

## Author Contributions

**Xinran Feng:** conceptualization, methodology, formal analysis, investigation, data curation, writing – original draft. **Ying An:** investigation, data curation, validation. **Fanfan Hou:** resources, methodology, technical support, data curation. **Qian Guo:** supervision, formal analysis, writing – review and editing. **Shanshan Cai:** supervision, validation, writing – review and editing. **Hongbin Xu:** supervision, conceptualization, writing – review and editing. **Youfu He:** supervision, project administration, writing – review and editing.

## Funding

The authors have nothing to report.

## Ethics Statement

The experiment involving human tissues was approved by the Ethics Committee of School of Stomatology, China Medical University (K2023007). The animal experiments were approved by the Ethics Commission of China Medical University, Shenyang, China (Approval No. CMU20231000).

## Consent

The authors have approved the manuscript for submission.

## Conflicts of Interest

The authors declare no conflicts of interest.

## Data Availability

The data that support the findings of this study are available from the corresponding author upon reasonable request.
